# Examining the Clinical Significance and Embryologic Development of Two Unique Cases of Renal Anatomy

**DOI:** 10.7759/cureus.74975

**Published:** 2024-12-02

**Authors:** Joseph Cherullo, Caleb Moffett, Yun Tan, Daniel T Daly

**Affiliations:** 1 Department of Surgery, Center for Anatomical Science and Education, Saint Louis University School of Medicine, Saint Louis, USA

**Keywords:** anatomy, collecting system, development, embryology, kidney, variation

## Abstract

Two unique presentations of renal anatomy were observed during routine cadaveric dissection. The first case presented with an ectopic malrotated left kidney supplied by supernumerary renal arteries. This kidney was drained by a circumaortic renal vein and an inferior polar vein. In addition to the vascular variations, this kidney had three extrarenal calyces and an anteriorly placed hilum. The second case presented with bilateral variations in renal anatomy. These variations included a left circumaortic renal vein, bilateral supernumerary arteries and veins, and a right testicular artery arising from an artery supplying the right kidney. These cases give insights into the high variability of renal anatomy, the clinical importance of atypical renal anatomy, and the embryological development of the renal system.

## Introduction

Knowledge and understanding of variations in renal anatomy provide opportunities to further understand both the embryologic development of the renal system and the clinical significance of atypical renal anatomy.

Each kidney is normally supplied by a single aortic branch, the renal artery, which divides into an anterior and posterior division. The anterior division further divides into four segmental arteries named apical, anterior superior, anterior inferior, and inferior, while the posterior division becomes the posterior segmental artery. Each kidney is also typically drained by one renal vein, which drains directly into the inferior vena cava. Large components of the collecting system of the kidney include two to three major calyces that merge to form the renal pelvis, which typically emerges from the renal hilum before narrowing to form the ureter [1].

Atypical renal arterial supply is a relatively common variation in renal anatomy. As a result, the arterial supply of each kidney is typically identified preoperatively using CT or MR imaging techniques [2]. For individuals with atypical arterial supply, the CT or MR images can be considered during preoperative planning to determine the best procedure for that specific individual. According to multiple authors, nearly 28% of kidneys are supplied by a renal artery and at least one additional atypical artery that arises from a different location [3]. Naming conventions for the atypical renal vasculature vary throughout the literature, though relatively common nomenclature describes all arteries that supply the kidney and the main renal artery as supernumerary arteries that can either be classified as accessory or aberrant. Accessory renal arteries enter the kidney at the renal hilum, while aberrant arteries bypass the renal hilum to supply the kidney by going through the renal parenchyma typically as polar arteries at the superior or inferior poles of the kidney [4].

Venous variations are reported far less frequently throughout the literature, and it is reported that 16.7% of kidneys are drained by at least one extra vein in addition to the renal vein. Cases of multiple renal veins are found more frequently on the right side (16.6%) than on the left side (2.1%) [5]. Extrarenal calyceal variations are also less frequently reported, with the literature reporting fewer than 60 cases [6]. The variations presented in these unique cases are likely all related to atypical embryologic development. These variations also can lead to clinical conditions such as posterior nutcracker syndrome, ureteropelvic junction obstruction, and hydronephrosis. Additionally, atypical renal anatomy can present challenges during surgical procedures such as kidney transplants or nephrectomies [7].

Case 1 was previously presented as a meeting abstract at the Anatomy Connected conference on March 25th, 2023. Case 2 was previously presented as a meeting abstract at the American Association of Clinical Anatomists annual meeting on July 9th, 2023.

## Case presentation

These donors were received through the CASE gift body program at Saint Louis University School of Medicine with signed informed consent and in accordance with all rules set forth by the Uniform Anatomical Gift Act.

Case 1

Case 1 presents a 78-year-old female cadaver with a listed cause of death of acute and chronic respiratory failure in addition to unspecified dementia. There was a self-reported health history of diabetes, depression, pain, and acute kidney failure among other issues.

**Figure 1:**
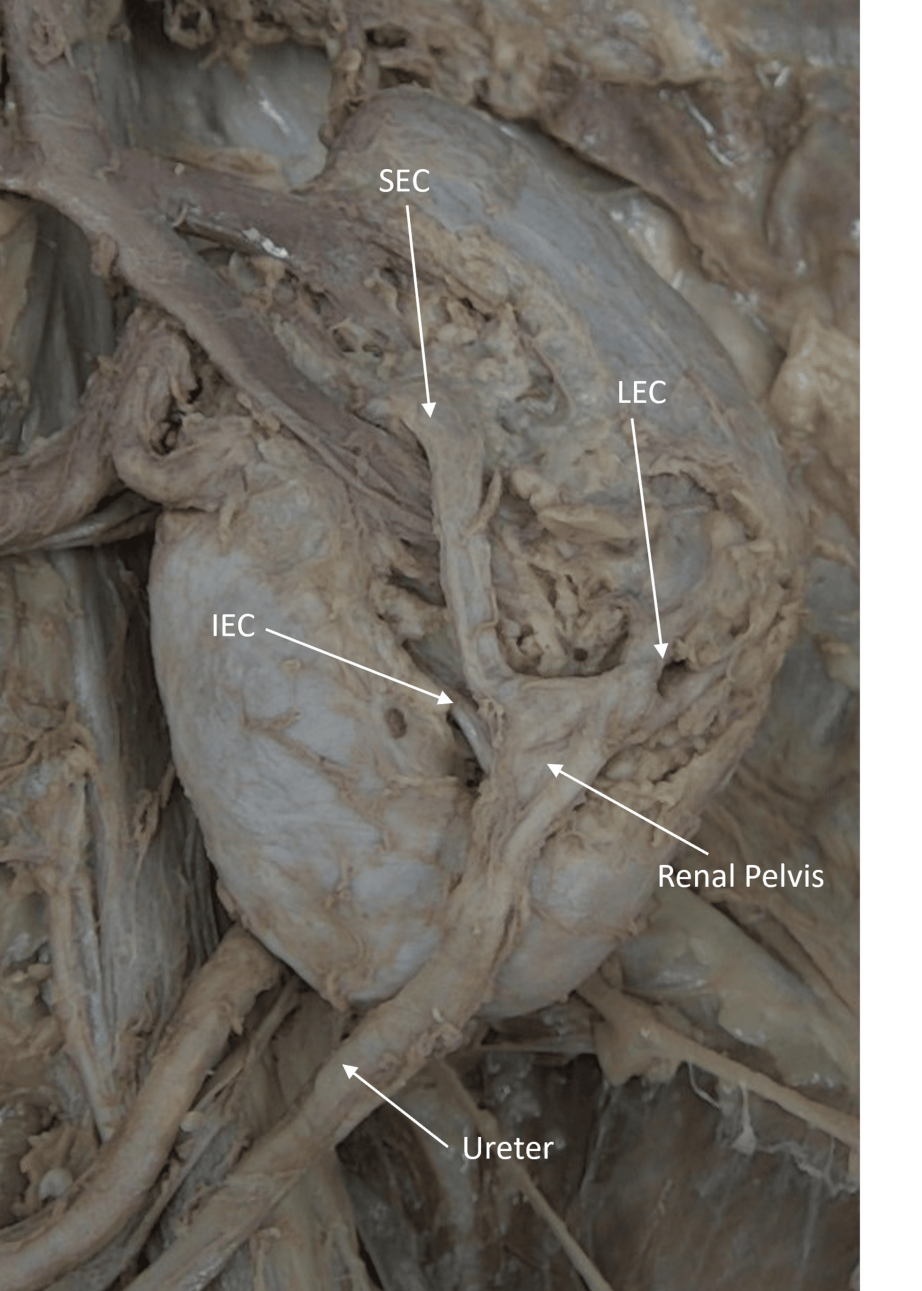
Case 1: Extrarenal calyces on the anterior surface Three extrarenal calyces are seen on the anterior surface of the malrotated left kidney. The calyces are named based on their position on the kidney’s surface. The superior (SEC), lateral (LEC), and inferior (IEC) extrarenal calyces all drain into the renal pelvis, which is also seen on the anterior surface. The renal pelvis continues as the ureter near the inferior pole of the kidney.

During routine cadaveric dissection, the left kidney was dissected from the perirenal fat. It was located just above the pelvic brim and noted to be rotated with portions of the collecting system and venous drainage emerging from the anterior renal surface. As the dissection continued, it was discovered that there were three separate portions of the collecting system outside the renal parenchyma draining into the renal pelvis, also found on the anterior surface. These were later named based on their position on the anterior surface of the kidney (superior, inferior, and lateral) extrarenal calyces (Figure 1).

**Figure 2:**
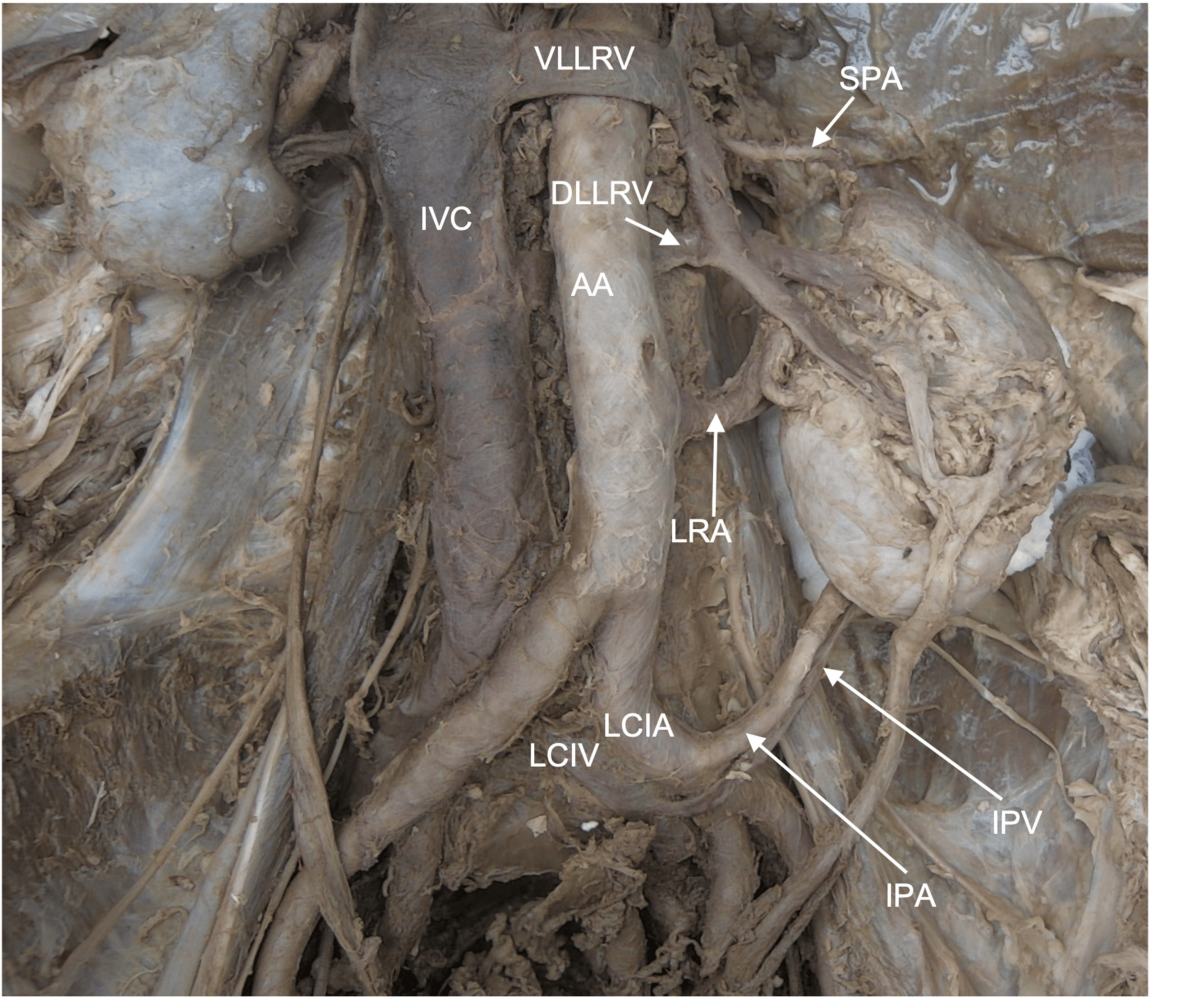
Case 1: Anterior surface and vasculature of the left kidney All three arteries supplying the left kidney can be observed. The superior polar artery (SPA) and inferior polar artery (IPA) are aberrant supernumerary arteries. SPA and the main left renal artery (LRA) both originate from the abdominal aorta (AA), while the IPA originates from the left common iliac artery (LCIA). Two veins are seen exiting the anterior surface of the kidney and draining into the circumaortic renal vein. The ventral (VLLRV) and dorsal (DLLRV) limbs of the circumaortic vein then drain into the inferior vena cava (IVC). An inferior polar vein (IPV) draining into the left common iliac vein (LCIV) is seen traveling with the same named artery.

**Figure 3:**
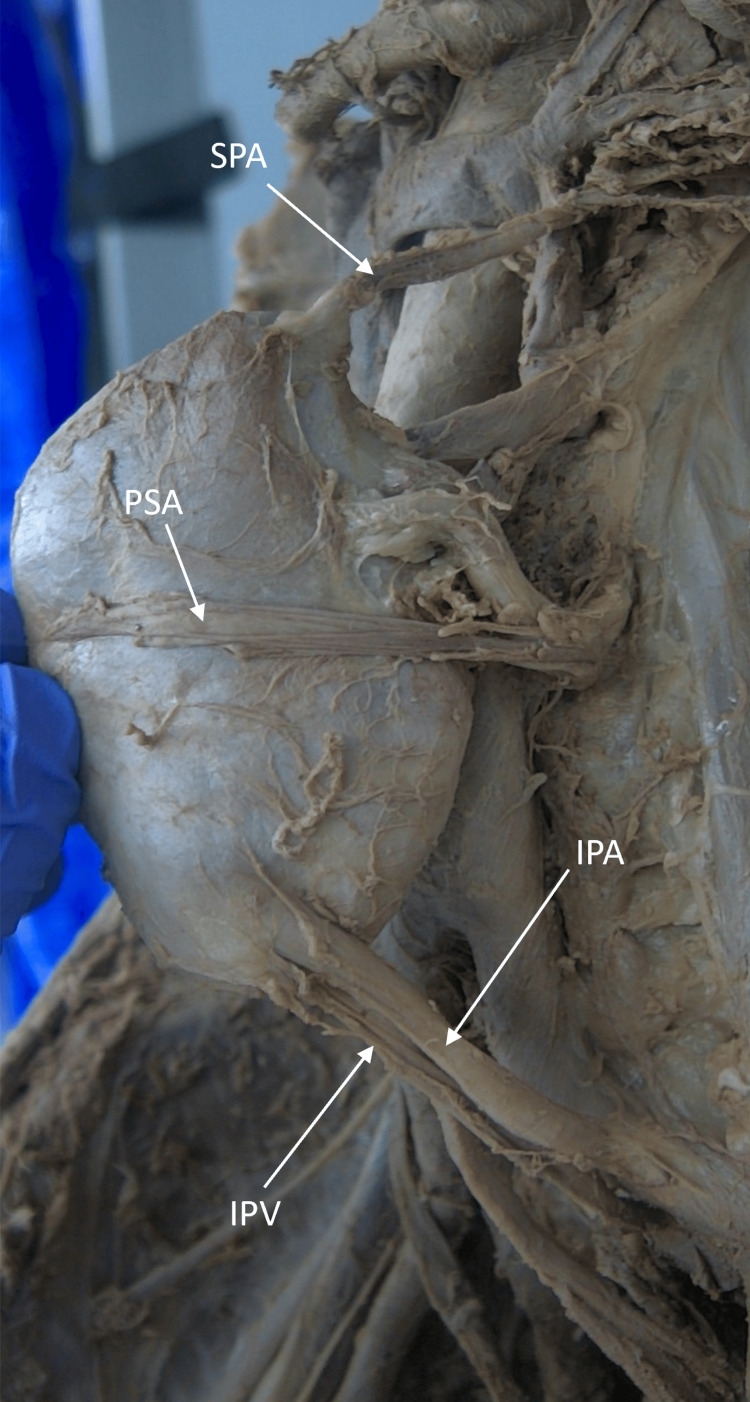
Case 1: Posterior surface of the left kidney The posterior surface of the left kidney shows the entry of the superior polar artery (SPA), posterior segmental artery (PSA), inferior polar artery (IPA), and inferior polar vein (IPV). The PSA, a branch of the main renal artery, traveled posteriorly on the renal surface until it entered the lateral border of the kidney. SPA is seen entering the superior pole of the kidney. The IPA and IPV are seen respectively supplying and draining the inferior pole of the kidney.

The two veins that emerged on the anterior surface formed a circumaortic renal vein, which drained into the inferior vena cava (Figure 2). Three branches coming from the main renal artery were observed entering the renal parenchyma. Two of these branches, the anterior superior and anterior inferior segmental arteries, entered through the medial wall (Figure 2). However, the third branch, the posterior segmental artery, traveled laterally on the posterior surface of the kidney before entering near the lateral border of the kidney (Figure 3).

The main renal artery was found coming off the lateral side of the abdominal aorta before dividing into anterior and posterior divisions 12.94 cm from its origin (Figure 2). The second artery supplying the kidney was identified as an inferior polar artery (IPA) that branched from the left common iliac artery (LCIA) and terminated on the posteroinferior border of the kidney (Figure 2). A vein connected to the left common iliac vein (LCIV) was found to be traveling with it and draining the inferior pole of the kidney (Figure 2). This vein was identified as the inferior polar vein (IPV), a supernumerary renal vein. The final artery supplying this kidney, a superior polar artery (SPA), was found. The SPA originated from the aorta and traveled posteriorly to the suprarenal gland to terminate at the posterosuperior border of the kidney (Figures 2, 3).

Upon completion of the dissection of the left kidney, it was observed that the right kidney’s renal artery branched into anterior and posterior divisions 17.54 mm from the aorta. Then the anterior division almost immediately divided into two branches, which further divided into both apical and anterior superior segmental arteries as well as anterior inferior and inferior segmental arteries near the renal hilum. The right kidney was also found to have a large cyst on its lateral surface.

The diameter of the renal arteries was then recorded bilaterally. On the left, measurements were taken with a digital caliper at the vessels’ origins. The main renal artery had the largest diameter of 0.534 cm. The IPA had the next largest diameter at 0.411 cm, and the superior polar was the smallest with a diameter of 0.263 cm. On the right, a single measurement was taken at the junction of the right renal artery (0.612 cm) with the abdominal aorta. The results for both the right and left kidneys are reported in Table 1.

**Table 1:** Case 1: Diameters of the renal arteries

Artery	Diameter (cm)
Left superior polar artery	0.263
Left main renal artery	0.534
Left inferior polar artery	0.411
Right main renal artery	0. 612

Next, to document the branching point of these renal arteries, the midpoint of the aortic hiatus was used as a reference point to locate the aortic origin of the SPA and the main renal artery. The measured distance was taken from the aortic hiatus to the superior border of the artery branching from the aorta. The most superior branch was the SPA branching 5.495 cm from the aortic hiatus. The main renal artery was the second branch originating 12.455 cm from the aortic hiatus. The aortic bifurcation, which was 16.040 cm from the aortic hiatus, was used as a reference point when measuring the location of the IPA. This artery originated 4.044 cm from the bifurcation. The right renal artery was measured the same way and found 4.921 cm from the aortic hiatus. The results for both kidneys are shown in Table 2.

**Table 2:** Case 1: Distance of the left and right renal arteries from aortic hiatus

Artery	Distance (cm)
Left superior polar artery	5.495
Left main renal artery	12.455
Left distance from aortic hiatus to aortic bifurcation	16.040
Left distance from aortic bifurcation to inferior polar artery	4.044
Right main renal artery	4.921

Renal vein diameter was measured using a digital caliper at the drainage point with the inferior vena cava (IVC) or common iliac vein. For the circumaortic renal vein, the diameter of each limb was recorded. The ventral limb, with a diameter of 1.509 cm, was considerably larger than the dorsal limb, which had a diameter of 0.841 cm. The IPV drained into the common iliac vein and had a diameter of 0.319 cm. The right renal vein had a diameter of 0.536 cm. As the veins were compressed, the diameter was calculated by adding the width and height of each vessel and then dividing the total by two. The diameters for each renal vein are reported in Table 3.

**Table 3:** Case 1: Diameters of the renal venous structures

Vein	Diameter (cm)
Ventral branch of circumaortic renal vein	1.509
Dorsal branch of circumaortic renal vein	0.841
Left inferior polar renal vein	0.319
Right renal vein	0.536

To document the location at which the renal veins drain into the IVC, the midpoint on the aortic hiatus was used again as a reference point. From this point, the superior border of the right renal vein and limbs of the circumaortic renal vein were measured. The ventral limb of the circumaortic vein was located 5.831 cm and the dorsal limb was located 9.416 cm from the aortic hiatus. The right renal vein was found to drain into the IVC 6.668 cm from the aortic hiatus. Finally, the IPV drained into the LCIV 3.754 cm from the bifurcation that occurred 18.108 cm from the aortic hiatus. These measurements are reported in Table 4, along with the measurements of the bifurcation of the inferior vena cava and the distance from that point to the inferior polar renal vein’s drainage point.

**Table 4:** Case 1: Distance of the renal venous structures from aortic hiatus to drainage point

Vein	Distance (cm)
Ventral branch of circumaortic renal vein	5.831
Dorsal branch of circumaortic renal vein	9.416
Bifurcation of the IVC from aortic hiatus	18.108
Inferior polar renal vein from bifurcation	3.754
Right renal vein	6.668

The extrarenal calyces were named superior, inferior, and lateral based on their location on the anterior surface of the kidney. The longest of these calyces was the superior, which traveled 2.945 cm before reaching the renal pelvis. The next longest was the lateral calyx, which traveled 1.626 cm, and the shortest was the inferior traveling only 0.795 cm. The diameter of the renal pelvis was 0.721 cm. The lengths that the three calyces traveled outside of the renal parenchyma to the renal pelvis are reported in Table 5.

**Table 5:** Case 1: Distance from the calyx to midpoint on renal pelvis

Calyx	Distance (cm)
Superior extrarenal calyx	2.945
Inferior extrarenal calyx	0.795
Lateral extrarenal calyx	1.626

Case 2

Case 2 presents a 75-year-old male cadaver. The donor’s self-reported medical history was unremarkable, and the cause of death was recorded as Alzheimer's disease and Parkinson’s.

Bilateral arterial and venous variations were observed in this donor. As the right kidney was dissected, three arteries supplying it were found to originate independently from the abdominal aorta. From superior to inferior, the first artery was a superior polar renal artery, entering the superior pole of the kidney on its anterior side, acting as a typical apical branch, which gave off the inferior suprarenal artery to supply the right suprarenal gland and a large, unusual branch running inferiorly along the posterior abdominal wall.

**Figure 4:**
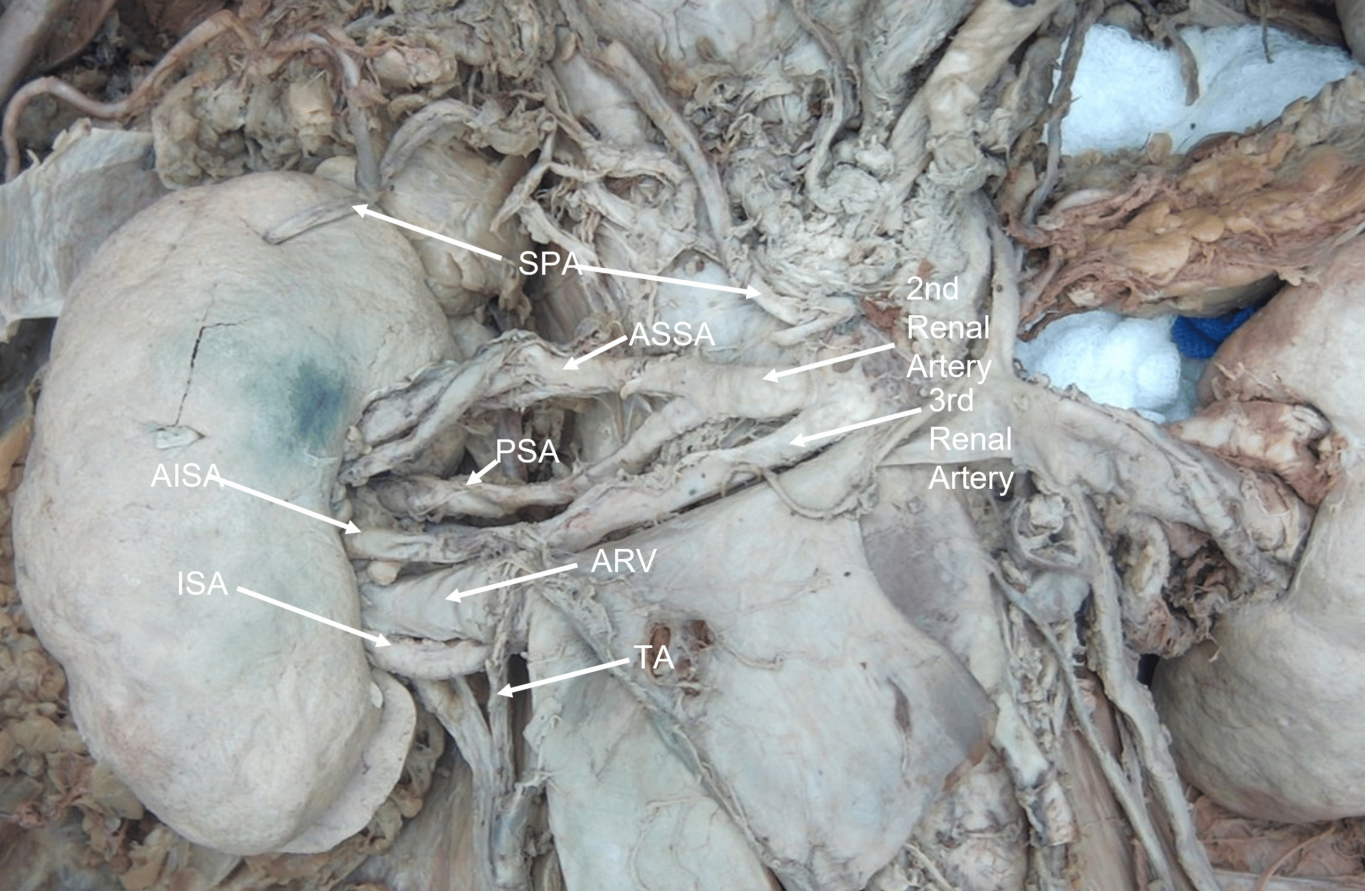
Case 2: Right kidney arterial supply Three arteries supplying the right kidney are shown in this image. The superior polar artery (SPA) and the second and third renal arteries all branch from the aorta. The anterior superior segmental artery (ASSA) and posterior segmental artery (PSA) branch from the second artery. The third artery trifurcates around the anterior renal vein (ARV) into the anterior inferior segmental artery (AISA), inferior segmental artery (ISA), and right testicular artery (TA).

**Figure 5:**
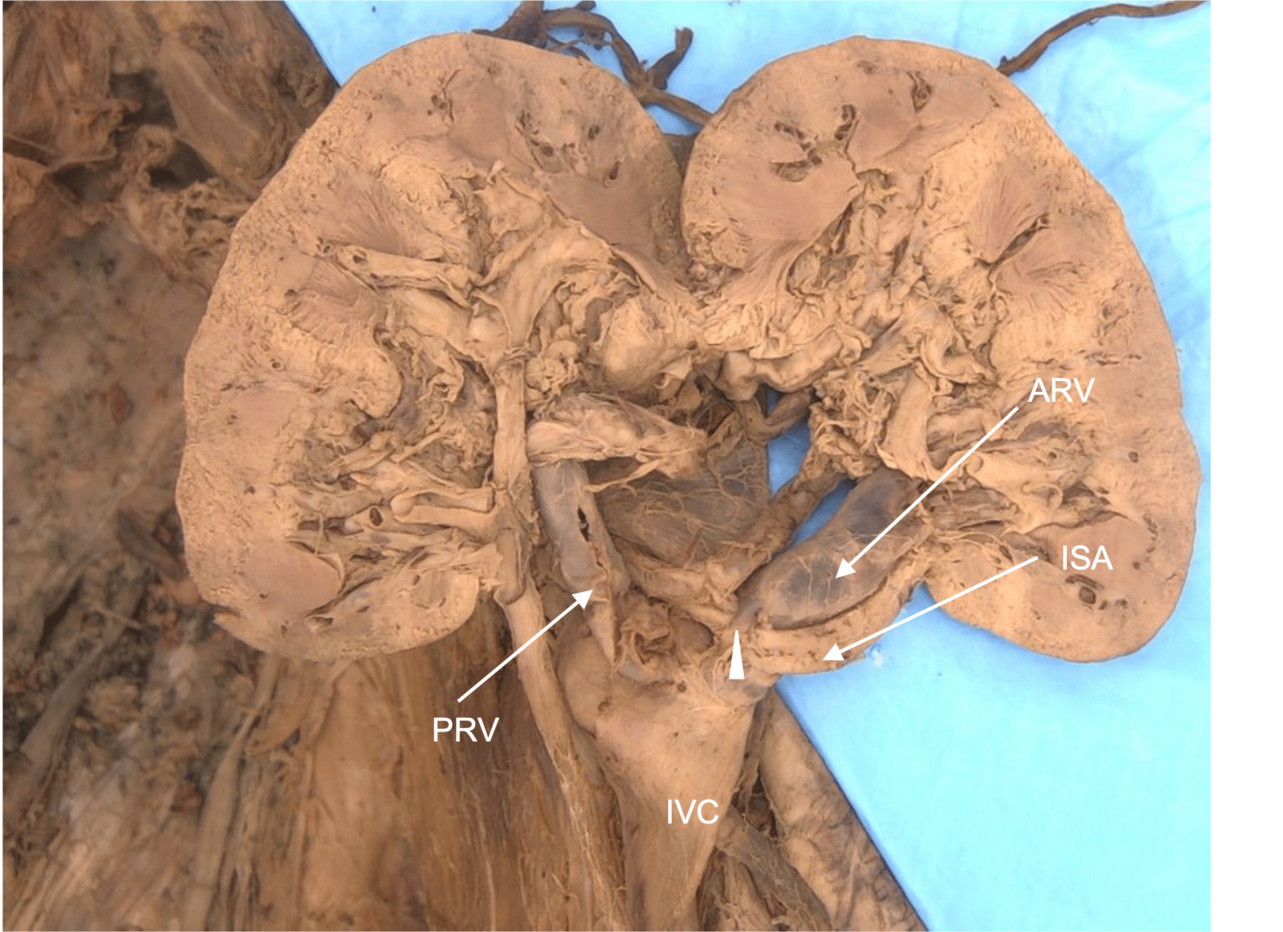
Case 2: Right kidney venous drainage (bisected) Anterior (ARV) and posterior renal veins (PRV) drain individually into the inferior vena cava (IVC). A segment of ARV securing the inferior segmental artery (ISA) is labeled with an arrowhead.

The second renal artery originated below the SPA. It divided quickly into the anterior superior segmental artery, which bifurcated into two smaller unnamed branches before reaching the hilum, and the posterior segmental artery (PSA), which entered the hilum posteriorly. The third artery branched immediately inferior to the second, trifurcating into the testicular, anterior inferior, and inferior segmental arteries. These two renal segmental arteries were separated by the right anterior renal vein, which traveled anteriorly to the vessels. The inferior segmental artery crossed the right anterior renal vein posteriorly and inferiorly along the posterior surface (Figures 4, 5). The vein had a small segment that traveled over the posterior surface of the inferior segmental artery before rejoining the anterior renal vein (Figure 5). This secured the inferior segmental artery to the posterior surface of the vein at this point. In addition to the anterior renal vein, a posterior renal vein emerging from the hilum drained into the IVC separately (Figure 5).

The diameter of each of the vessels was measured using a digital caliper at the junction of the aorta and from the branch point for the segmental arteries. The SPA, with a diameter of 0.249 cm, was the smallest of the three direct branches from the aorta. The second renal artery with a diameter of 0.660 cm was the largest. The anterior superior segmental artery (ASSA) with a diameter of 0.473 cm and PSA with a diameter of 0.342 cm were branches of the second artery. The third renal artery was considerably smaller with the diameter only being 0.342 cm. It trifurcated into the testicular, anterior inferior segmental artery (AISA) with a diameter of 0.398 cm and inferior segmental artery (ISA) with a diameter of 0.391 cm. The diameters of the right renal veins were recorded as well. The anterior renal vein (ARV) was slightly smaller with a diameter of 0.782 cm than the posterior renal vein (PRV), which has a diameter of 0.807 cm. These measurements are summarized in Table 6.

**Table 6:** Case 2: Diameters of all vessels supplying or draining the right kidney

Blood Vessel	Diameter (cm)
Superior polar artery	0.249
2nd renal artery	0.660
3rd renal artery	0.391
Anterior superior segmental artery	0.473
Posterior segmental artery	0.342
Anterior inferior segmental artery	0.398
Inferior segmental artery	0.391
Anterior renal vein	0.782
Posterior renal vein	0.807

The distance of each vessel from the aortic hiatus to the superior border of the vessel's lumen was recorded using the same digital caliper. The three right renal arteries identified from superior to inferior were superior polar, second renal, and third renal. The distance of each of these arteries’ origin from the aortic hiatus was 6.311, 7.014, and 7.595 cm, respectively. The ARV was found 8.640 cm from the aortic hiatus, while the posterior was found 7.780 cm from the aortic hiatus. The results are summarized in Table 7.

**Table 7:** Case 2: Distance of right renal vessels from the aortic hiatus to origin or drainage point

Blood Vessel	Distance (cm)
Superior polar artery	6.311
2nd renal artery	7.014
3rd renal artery	7.595
Anterior renal vein	8.640
Posterior renal vein	7.780

**Figure 6:**
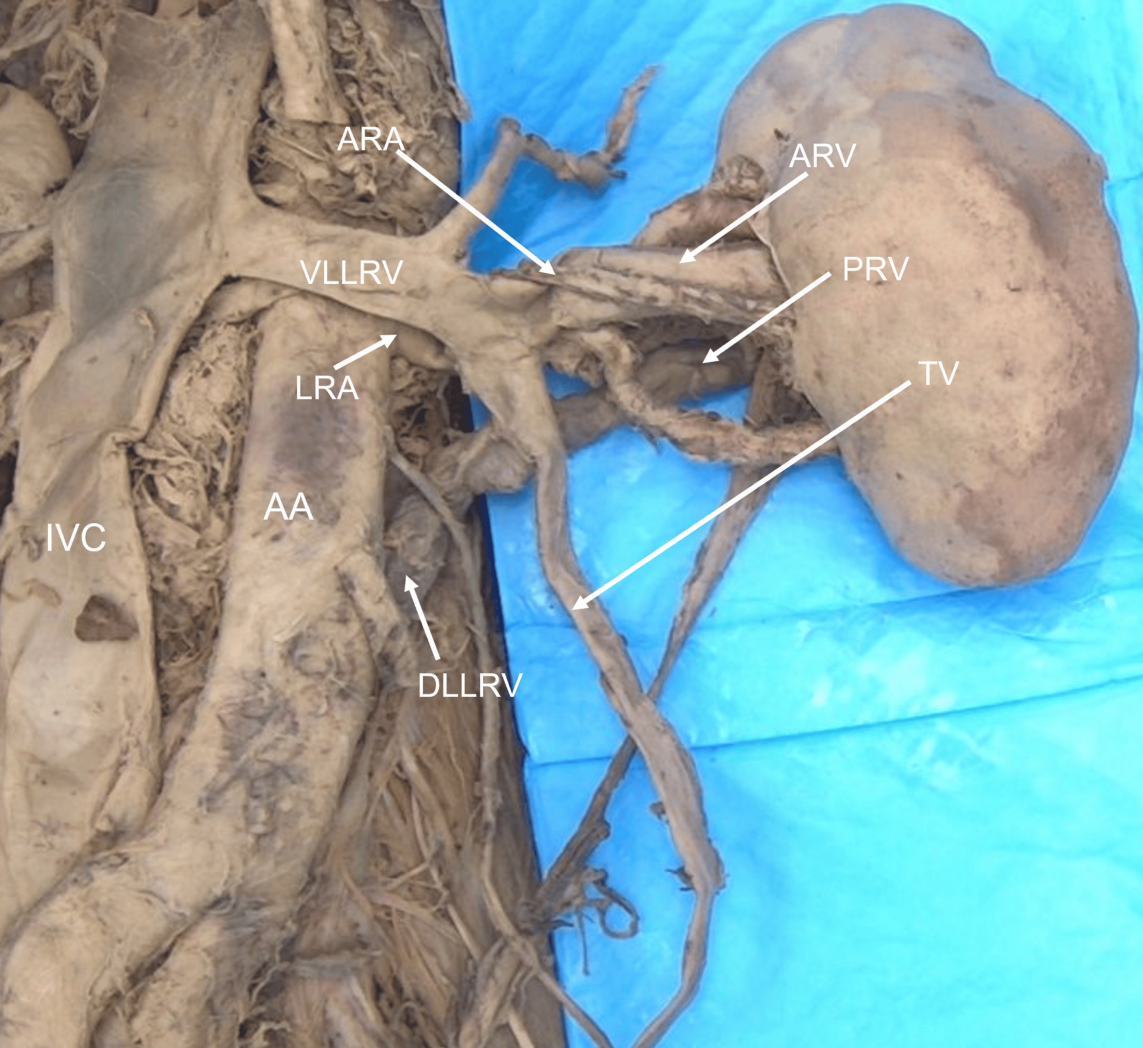
Case 2: Left kidney anterior surface vasculature The left main renal artery (LRA) and accessory renal artery (ARA) are seen both branching from the aorta (AA). An anterior renal vein (ARV) and posterior renal vein (PRV) form a circumaortic vein. The ventral (VLLRV) and dorsal limbs (DLLRV) of the circumaortic vein are seen draining into the inferior vena cava (IVC). The testicular vein (TV) is seen draining into both limbs of the circumaortic vein.

**Figure 7:**
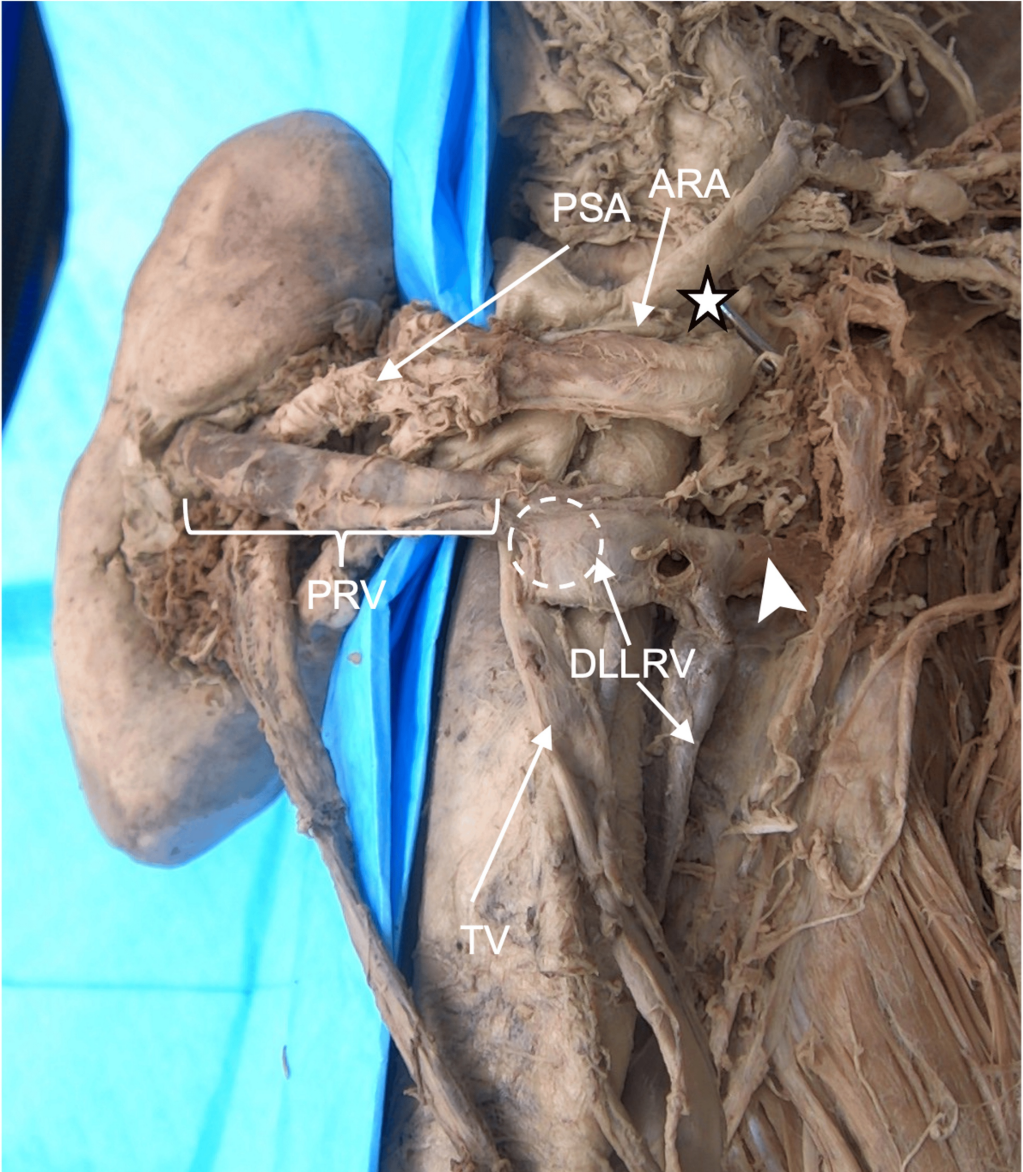
Case 2: Left kidney posterolateral surface The connection from the dorsal limb of the circumaortic vein to the ascending lumbar vein is indicated by the arrowhead. The posterior segmental artery (PSA) is seen entering the hilum. The dashed circle shows the location of the testicular vein (TV) draining into the dorsal limb (DLLRV) of the circumaortic renal vein. This junction is also the point where the DLLRV begins and the PRV ends. The posterior renal vein (PRV) is seen emerging from the renal hilum. The star represents the origin of the aorta for the accessory renal artery (ARA).

The left kidney was found to have two arteries (Figure 6). The first was anatomically typical and divided into anterior and posterior divisions, with the anterior division ultimately giving rise to the apical, anterior superior, and inferior segmental arteries, and the posterior division continued as the PSA. An accessory artery was found superior to the main renal artery. This accessory renal artery traveled around the ARV to its anterior side before ultimately terminating at the typical location of an AISA (Figures 6, 7). The left renal vein was observed to be circumaortic (Figures 6, 7). The ventral limb was found inferior to the superior mesenteric artery, and the dorsal limb was found near the level of the bifurcation of the aorta (Figure 6). The retro aortic (dorsal) portion and ventral portions were connected via the left testicular vein (Figures 6, 7). The dorsal limb also had a connection to the ascending lumbar vein (Figure 7). Two veins emerged from the renal hilum. The first emerged anteriorly and was named the ARV. The second vein appeared to be draining the posterior surface, which was named the PRV.

The diameters of the left kidney's vessels were measured as well. The accessory and main renal arteries were found to have diameters of 0.214 and 0.673 cm, respectively. The anterior division of the main artery with a diameter of 0.537 cm is slightly larger than the posterior division with a diameter of 0.476 cm. These data are summarized in Table 8.

**Table 8:** Case 2: Diameters of all vessels supplying or draining left kidney

Blood Vessel	Diameter (cm)
Accessory artery	0.214
Main renal artery	0.673
Anterior division	0.537
Posterior division	0.476
Ventral limb circumaortic renal vein	0.534
Dorsal limb circumaortic renal vein	0.585

Using the same procedure as before, the distance from the aortic hiatus to each vessel’s origin or drainage point was recorded. The accessory artery and main renal artery branched off the aorta at 6.183 cm and 6.690 cm from the aortic hiatus, respectively. The ventral and dorsal limbs each drained into the vena cava at 6.840 and 13.680 cm from the same reference point. These results are shown in Table 9.

**Table 9:** Case 2: Distance of the left renal vessels from the aortic hiatus to the origin

Blood Vessel	Distance (cm)
Accessory artery	6.183
Main renal artery	6.690
Ventral limb circumaortic renal vein	6.840
Dorsal limb circumaortic renal vein	13.680

## Discussion

From the review of the literature, it can be concluded that the collection of variations present in each of these two cases of renal anatomy has not been reported previously and is unique anatomically. A comparison of the current cases with a selection of previously published cases containing combinations of venous, arterial, and collecting system variations is presented in Table 10 [8-12]. While none of the individual variations in the current cases are novel by themselves, the unique combinations allow for a detailed exploration of the embryologic development of the urogenital system and can guide discussion around why atypical renal anatomy is clinically relevant.

**Table 10:** Selection of reported cases with combinations of renal arterial, venous, and collecting system variations

Authors	Arterial Variation	Venous Variation	Collecting System Variation
Zăhoi et al. [8]	• Three left renal veins		N/A
	• All from aorta	• Three left renal veins	
	• Superior polar	• Typical left renal vein draining into IVC	
	• Main renal	• Middle renal vein draining into the left gonadal vein	
	• Inferior polar sharing trunk with median sacral	• Left and right inferior renal veins (polar veins) join before draining into the left common iliac vein	
	• Two right renal arteries	• Two right renal veins	
	• Both from aorta	• Typical right renal vein draining into IVC	
	• Main renal	• Right and left inferior renal veins (polar veins) join before draining into the left common iliac vein	
	• Inferior polar near aortic bifurcation		
Bachul et al. [9]	• Five left renal arteries	N/A	Short duplication of ureter
	• Four from aorta		
	• Two entering superior pole		
	• Two entering hilum		
	• Inferior polar from left common iliac		
Orlando et al. [10]	• Six right renal arteries	N/A	Double renal pelvis draining into single ureter
	• All branching from aorta		
	• 1st branch superior polar branches 2-5 entered the hilum		
	• 6th branch inferior polar		
Gupta et al. [11]	• Three right renal arteries	Renal veins traveled with arteries (no further elaboration)	Four extrarenal calyces and renal pelvis outside the renal parenchyma
	• Two from aorta		
	• One from common iliac		
Ahuja et al. [12]	• Two left renal arteries	• Circumaortic renal vein	Three extrarenal calyces
	• Both branching from aorta	• Draining into IVC	
Case 1	• Three left renal arteries	• Circumaortic renal vein	Three extrarenal calyces on the anterior surface of the kidney
	• Two from aorta	• Draining into IVC	
	• Superior polar	• Inferior polar renal vein	
	• Main renal	• Draining into the left common iliac	
	• Inferior polar from the left common iliac		
Case 2	• Two left renal arteries	• Circumaortic renal vein	N/A
	• Both from aorta	• Draining into IVC	
	• Accessory	• Two right renal veins	
	• Main renal	• Draining into IVC separately	
	• Three right renal arteries		
	• All from aorta		
	• Superior polar artery		
	• Two arteries entering hilum		

It is reported in the literature that 28.2% of kidneys are supplied by more than one renal artery [3]. This indicates that the presence of one or more supernumerary renal arteries supplying a kidney, similar to the one in the cases presented above, is not uncommon. These supernumerary arteries typically originate from the aorta. Cases of an IPA branching from the left common iliac, observed in Case 1, have not been frequently reported in the literature, making it a rarer and significant variation.

Historically, Felix’s ladder theory provided the widely accepted answer to how supernumerary renal arteries occur during development. He reported nine pairs of lateral mesonephric arteries arising from the dorsal aorta that supply the mesonephric kidney and surrounding tissues. The nine pairs are divided into three different groups: the first and second are cranial, the third through fifth are middle, and the sixth through ninth are caudal. He hypothesized that the middle group gave rise to the permanent renal arteries and that the presence of multiple renal arteries was the result of a persistent mesonephric artery from the middle group [13].

Recent studies have found that these mesonephric arteries regress completely before permanent renal arteries appear [14]. It has also been discovered that the permanent and supernumerary arteries branch from the dorsal aorta during the ascent of the metanephric kidney during development [15]. The IPA seen in Case 1 could be a persistent primary renal artery. The primary renal artery is seen branching from the common iliac artery and supplies the metanephric kidney before its ascent [15].

Gonadal arteries branching from a renal artery, such as the right testicular artery found in Case 2, have been discussed in the literature. This branching pattern has been found in up to 18% of individuals [16]. Additionally, gonadal artery variations are also linked to the embryologic development of the urogenital system, and variations in renal anatomy are frequently observed with them [16].

Renal venous variations are reported less frequently. A recent meta-analysis using data from 105 published articles found that 3.5% of individuals possess a left circumaortic renal vein as seen in Cases 1 and 2. This same meta-analysis found that 16.7% of kidneys were drained by multiple veins. More specifically, 16.6% of right kidneys and 2.1% of left kidneys were drained by multiple veins [5]. This indicates that the two veins draining the left kidney in Case 2 are significantly more uncommon than the anterior and PRVs seen draining the right kidney. The case of an IPV draining into the LCIV as seen in Case 1 has been rarely reported in the literature [17].

An examination of how the left and right renal veins develop embryologically explains how the variations, which are seen in both reported cases, occur. The renal venous system develops from a series of new connections between the right and left posterior cardinal, subcardinal, and supracardinal veins [18]. Supernumerary veins draining the right kidney are believed to be the result of the failure of the previously mentioned cardinal veins to merge properly [18]. The embryologic development of the left renal vein is more complex. The left renal vein forms from the anastomosis between specific pairs of the cardinal veins, forming a circumaortic ring. The dorsal limb of the ring is typically obliterated, leaving the ventral limb to persist as the left renal vein [19]. When the dorsal limb fails to regress, the circumaortic renal vein will be found draining the left kidney in adults. The size of the collar formed by the ventral and dorsal limbs is variable. This is because the length of the dorsal limb varies. The left supracardinal vein is understood to be the dorsal limb in the embryonic circumaortic renal vein, and the more of the vein that persists, the larger the collar [20].

Cases of extrarenal calyces, as seen in Case 1, have rarely been discussed in the published literature. Less than 60 total cases have ever been reported, and most of the cases have been discovered postmortem [6]. An extrarenal calyx is found outside of the renal parenchyma and can include the major and/or minor calyces. This variation is often found in association with other kidney abnormalities such as renal malrotation and ectopia [21]. This association is observed in Case 1. The left kidney with extrarenal calyces had an anterior-facing renal hilum and was located near the pelvic brim.

The exact cause of extrarenal calyces is unknown. However, in cases of malrotation and ectopic location, it has been hypothesized to be the result of the ureteric bud failing to properly indent the nephrogenic mass. As a result, the developing metanephric kidney will not receive the stimulus required to start or complete its ascend out of the pelvis, and it will not rotate properly [11].

All the atypical anatomy found in these two cases is related to atypical embryologic development of the urogenital system. The numerous variations in each case may be related to the altered function of primary cilia, which act as specialized sensory organelles that play important roles in tissue development and signal transduction. Mutations in these cells manifest as renal ciliopathies characterized by kidney dysfunction related to pathologies such as polycystic kidney disease, nephronophthisis, and Bardet-Biedl syndrome [22]. While none of these diseases appear to have affected either individual case presented in this article, cilia are critical in the embryological development of “sidedness” [23], and unilateral renal variations may be the result of altered cilia function.

One specific reason that altered primary cilia function could affect renal anatomy and function is that they are involved in the Wnt signaling pathway [24]. One example of this is the expression of WNT1 in the metanephric mesenchyme. This expression allows for the tissue within the metanephric blastema to respond to the induction of the ureteric bud [25]. As stated previously, it is believed that failure of the ureteric bud to properly impact and signal the metanephric blastema could be the cause of variations such as extrarenal calyces, ectopia, and malrotation.

However, if the primary cilia involved in this signaling pathway are damaged, proper impact by the ureteric bud will not be relevant because the signal pathway is already compromised. Thus, altered primary cilia function could be just as likely to cause cases of ectopia, malrotated kidney, and extrarenal calyces.

Clinical significance

Knowledge of common and uncommon variations present in a given individual’s renal anatomy will be significant during surgical procedures in the abdomen. Nephrectomy or partial nephrectomy would require a surgeon to know what vessels are supplying or draining each specific lobe of the kidney. Additionally, the presence of supernumerary arteries or veins would create a challenge during kidney transplants. Each vessel that needs to be disconnected in the donor and reconnected in the recipient is another variable that needs to be accounted for during the procedure. Typically, renal vessels are screened clinically using techniques such as computed tomography angiography and magnetic resonance angiography as well as renal duplex ultrasound [26]. If the presence of these atypical vessels is unknown, it can lead to accidents and complications. Ectopic location and malrotation of a kidney as seen in Case 1 must also be accounted for during surgical procedures to avoid complication.

**Figure 8:**
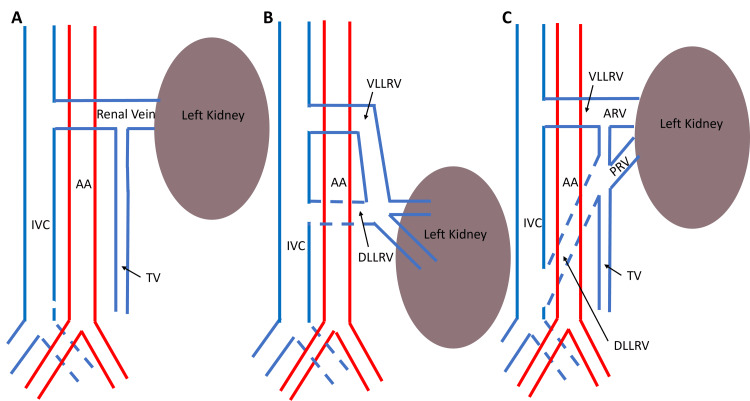
Simple schematic showing typical left venous drainage (a), circumaortic vein for case 1 (b), and case 2 (c) Arteries are shown in red, and veins are shown in blue in these schematics comparing typical anatomy (A) and the two cases of renal vascular variations (B and C). The abdominal aorta (AA) is located to the left of the inferior vena cava (IVC). In both cases, the ventral (VLLRV) and dorsal (DLLRV) limbs of the circumaortic renal vein drain into the IVC. In case 1, the circumaortic renal vein receives two veins emerging from the anterior surface of the left kidney. In case 2, an anterior (ARV) and posterior renal vein (PRV) drain into the circumaortic renal vein.

In cases of circumaortic renal veins, knowledge of the varying lengths of the dorsal limbs would be significant in some surgical procedures in the abdomen. The variability is observable in Cases 1 and 2 presented in this article. In Case 1, the dorsal limb drains into the IVC 0.66 cm below the ventral limb. However, in Case 2, the dorsal limb drained into the IVC 6.84 cm below the ventral limb and was near the junction of the common iliac veins. This difference is illustrated in Figure 8.

Posterior nutcracker syndrome can occur in individuals with circumaortic venous drainage due to the compression of the dorsal limb. This compression can result in increased venous pressure, which can lead to hematuria and congestion of the kidney [27].

Supernumerary arteries have been reported to be associated with the presence of hypertension [28]. Whether this is a causal relationship remains unclear, but one hypothesis for the mechanism involved is the small diameter of supernumerary arteries that may result in decreased perfusion pressure and increased blood flow resistance in the renal parenchyma, resulting in stimulation of the renin-angiotensin-aldosterone pathway that leads to an increase in blood pressure [28].

Ureteropelvic junction obstruction can also occur due to atypical renal anatomy, specifically in cases with circumaortic renal vein or IPA because the atypical vessel crosses directly over the renal pelvis or ureter [29]. In severe cases, this can lead to hydronephrosis.

Cases of extrarenal calyces can create a false impression of hydronephrosis since the atypical anatomy can become distended during retrograde pyelography [11]. However, from the review of the literature, it appears that the extrarenal calyx variation may not have a negative impact on renal function. As a result, many cases may go undiscovered clinically, likely contributing to the lack of reported cases. The variation seems to be difficult to identify preoperatively; therefore, surgeons should be aware of its potential presence when performing procedures on the kidney itself.

## Conclusions

Both unique cases of atypical renal anatomy presented here provide an opportunity to explore the different variations in renal anatomy that can occur. Additionally, these cases offer insights into why knowledge of common and uncommon variations in renal anatomy is clinically relevant. For example, in the first case, understanding the left kidney’s ectopic location and atypical vascularization would be critical for surgeons operating on the left side of the abdomen or pelvis. In the second case, the supernumerary vessels supplying or draining each kidney would pose challenges during nephrectomy. Lastly, the numerous variations in both cases help to understand how the kidneys develop embryologically. The IPA in Case 1 supports the idea that the metanephric kidney is originally supplied by a primary renal artery in the pelvis before completing its ascent out of the abdomen. It is possible that an IPV could be a primary renal vein that did not regress. Additionally, the circumaortic renal veins in both cases illustrate the variability in the length of the dorsal limb, which is the persistent left supracardinal vein.
